# Spatiotemporal Assessment of COVID-19 Spread over Oman Using GIS Techniques

**DOI:** 10.1007/s41748-020-00194-2

**Published:** 2020-12-08

**Authors:** Khalifa M. Al-Kindi, Amira Alkharusi, Duhai Alshukaili, Noura Al Nasiri, Talal Al-Awadhi, Yassine Charabi, Ahmed M. El Kenawy

**Affiliations:** 1 Geography Department, Sultan Qaboos University, Muscat, Oman; 2 Physiology Department, Colege of Medicine and Health Sciences, Sultan Qaboos University, Muscat, Oman; 3 Department of Geography, Mansoura University, Mansoura, 35516 Egypt; 4 University of Technology and Applied Sciences, Nizwa, Oman; 5 Center for Environmental Studies and Research, Geography Department, Sultan Qaboos University, Muscat, Oman

**Keywords:** COVID-19, GIS, Moran’s *I*, Oman, $G_{i}^{*}$, Spatial analysis

## Abstract

Coronavirus disease (COVID-19), caused by acute respiratory syndrome coronavirus 2 (SARS-CoV-2), is a worldwide challenge effecting millions of people in more than 210 countries, including the Sultanate of Oman (Oman). Spatiotemporal analysis was adopted to explore the spatial patterns of the spread of COVID-19 during the period from 29th April to 30th June 2020. Our assessment was made using five geospatial techniques within a Geographical Information System (GIS) context, including a weighted mean centre (WMC), standard deviational ellipses, Moran’s *I* autocorrelation coefficient, Getis-Ord General-G high/low clustering, and Getis-Ord $G_{i}^{*}$ statistic. The Moran’s *I*-/*G*- statistics proved that COVID-19 cases in datasets (numbers of cases) were clustered throughout the study period. The Moran’s *I* and *Z* scores were above the 2.25 threshold (a confidence level above 95%), ranging from 2274 cases on 29th April to 40,070 cases on 30th June 2020. The results of $G_{i}^{*}$ showed varying rates of infections, with a large spatial variability between the different wilayats (district). The epidemic situation in some wilayats, such as Mutrah, As-Seeb, and Bowsher in the Muscat Governorate, was more severe, with *Z* score higher than 5, and the current transmission still presents an increasing trend. This study indicated that the directional pattern of COVID-19 cases has moved from northeast to northwest and southwest, with the total impacted region increasing over time. Also, the results indicate that the rate of COVID-19 infections is higher in the most populated areas. The findings of this paper provide a solid basis for future study by investigating the most resolute hotspots in more detail and may help decision-makers identify targeted zones for alleviation plans.

**Supplementary Information:**

The online version contains supplementary material available at 10.1007/s41748-020-00194-2.

## Introduction

Coronavirus disease 2019 (COVID-19), the recent greatest threats encountering the globe, has been declared as a pandemic by the World Health Organization (WHO) since March 2020. The ongoing global interest of this massive health risk is motivated mainly by the accelerated rate of spread this pandemic, besides its substantial health, socioeconomic, and even political consequences over both developed and developing countries (Torales et al. 2020; Coccia 2020). According to the WMO Covid-19 dashboard (https://covid19.who.int/), to date (17th November), the number of confirmed cases across the globe exceed 55 million, while deaths approach 1.4 million (Riou and Althaus 2020). According to Cutler and Summers. (2020), the estimated costs of the COVID-19 pandemic in the US may reach $16 trillion (approximately 90% of the annual gross domestic product), exceeding the cost of the Iraq War and approaching the costs of global climate change (Ficetola and Rubolini 2020).

Much efforts have been made to control the spread of the COVID-19 at local, national, and global scales (Chakraborty and Maity 2020). However, the ‘global’ strategy to cope with this emerging pandemic has been hampered by many challenges, primarily its ‘creeping’ nature and rapid transmission rate, which to date has impacted more than 210 countries and territories worldwide (Nicola et al. 2020; Sohrabi et al. 2020; Bourgonje et al. 2020). Several studies have confirmed its extraordinary rate of transmission, associated with certain socioeconomic and environmental factors (Shereen et al. 2020; Gupta et al. 2020; Li et al. 2020; Saadat et al. 2020). Amongst these efforts, many researchers worldwide have been attempting to understand the behavior of this pandemic, particularly its transmission, detection, treatment and socioeconomic impacts (Allington et al. 2020; Alzamora et al. 2020; Liu et al. 2020a,b; Gross et al. 2020; Elmousalami and Hassanien 2020).

The spread of diseases in general and infectious diseases in particular is inevitably spatial. Public health experts can identify how infections move via local or even global transmission by following contact trajectories within population networks (Salinsky and Gursky 2006; Mackey et al. 2014). In this regard, Geographical Information System (GIS) is a powerful analytical tool, not only coz it incorporates fundamental epidemiological information on humans, times and locations but also coz it acts as a shared interface for centralised reporting and tracking of indicators from various areas (e.g. epidemiologic data georeferencing) (Law and Wilfert 2004; Esri 2011). These advantages allow epidemiologists produce maps showing the spatial distribution of diseases at various scales: global, regional, national, provincial or local. These maps enable scientists to better predict which populations will be vulnerable and their levels of exposure to the risk of infections. Also, probabilistic risk maps at detailed spatial scales allow epidemics to be tracked, strategies for prevention and control to be prioritised, and local authorities to assign appropriate budgets for disease control. In addition, GIS makes the propagation of infectious disease easier to visualise by temporary map animation and network analysis (Boulos and Geraghty 2020; Zhou et al. 2020). With the aid of information technology (IT) solutions, improved accuracy, efficiency, resource monitoring and cost savings can support sound and significant investments across the entire public health sector (Kittayapong et al. 2008; Allam and Jones 2020). Numerous studies have provided evidences that, as part of a hospital or emergency operations centre, GIS is an essential tool for many situational awareness programs dealing with pandemic diseases (Sithiprasasna et al. 2004).

Understanding the spatiotemporal incidences of COVID-19 at the national level is extremely important to deliver vital perspicacity into how epidemics occur, continue, and recede. Oman is one of the world countries facing the pandemic risk of COVID-19, with its first confirmed case registered on 28th February 2020 in Muscat. According to the Omani Ministry of Health, the infection rate has increased sharply since the late of April to the mid of August, with almost 2274–82,924 confirmed cases over this period, with a broader distribution in the majority of wilayats. From the demographic perspective, Oman currently represents one of the top-ranking countries in terms of the percentage of confirmed cases to the total population of the country, with almost 23,000 cases per million (https://covid19.who.int/; mid-November 2020). Although many studies have employed spatial techniques to assess spatial and temporal characteristics (e.g. centre, density, hotspots, cold spots, direction, etc.) COVID-19 (e.g. Biswas and Sen 2020; Danon et al. 2020; Kang et al. 2020; Zhou et al. 2020), no research to yet was found for Oman. Such an assessment is important to quantify spatial and temporal patterns of COVID-19 spread, assess its track changes over time, and determine the different demographic, environmental, and socioeconomic variables that may accelerate transmission and infection rates. From a policy standpoint, this assessment is desired to aid policy makers develop their plans and strategies in a more reliable way, taking into consideration spatial variations of this health massive threat. In this context, GIS—through a wide variety of spatial statistics—can play a significant role in delineating the spatial and temporal patterns of COVID-19 in Oman. Specifically, advancements in GIS techniques, particularly spatial modeling and data mining, have made it is possible to provide an inclusive picture of the primary spatial hotspots of this virus at different spatial scales (e.g. Wang et al 2020; Kamel Boulos and Geraghty 2020; Sarwar et al. 2020; Mollalo et al. 2020; Bherwani et al. 2020; Zhu and Xie 2020; Adekunle et al. 2020; Shi et al. 2020). A representative example is Ramírez-Aldana et al. (2020) who applied spatial statistics to characterize COVID-19 patterns over Iran.

The main aims of this study are to (1) assess the spatiotemporal patterns of COVID-19 spread using data of confirmed cases, which varied from 2274 (29th April) to 40,070 (30th June 2020); (2) quantify temporal variations of the rate of infection; and (3) compare variations in the daily infection rate at wilayat level. Results of this can contribute to understanding the dynamics and processes controlling the spread of COVID-19 over both space and time, which can help policy makers adopt more appropriate actions and strategies to mitigate the spread of this pandemic in Oman. Herein, it should be indicated that this study uses the term “wilayat”, which is a local administrative level, equal to a “county” in the U.S.

## Materials and Methods

### Study Area

**Fig. 1:**
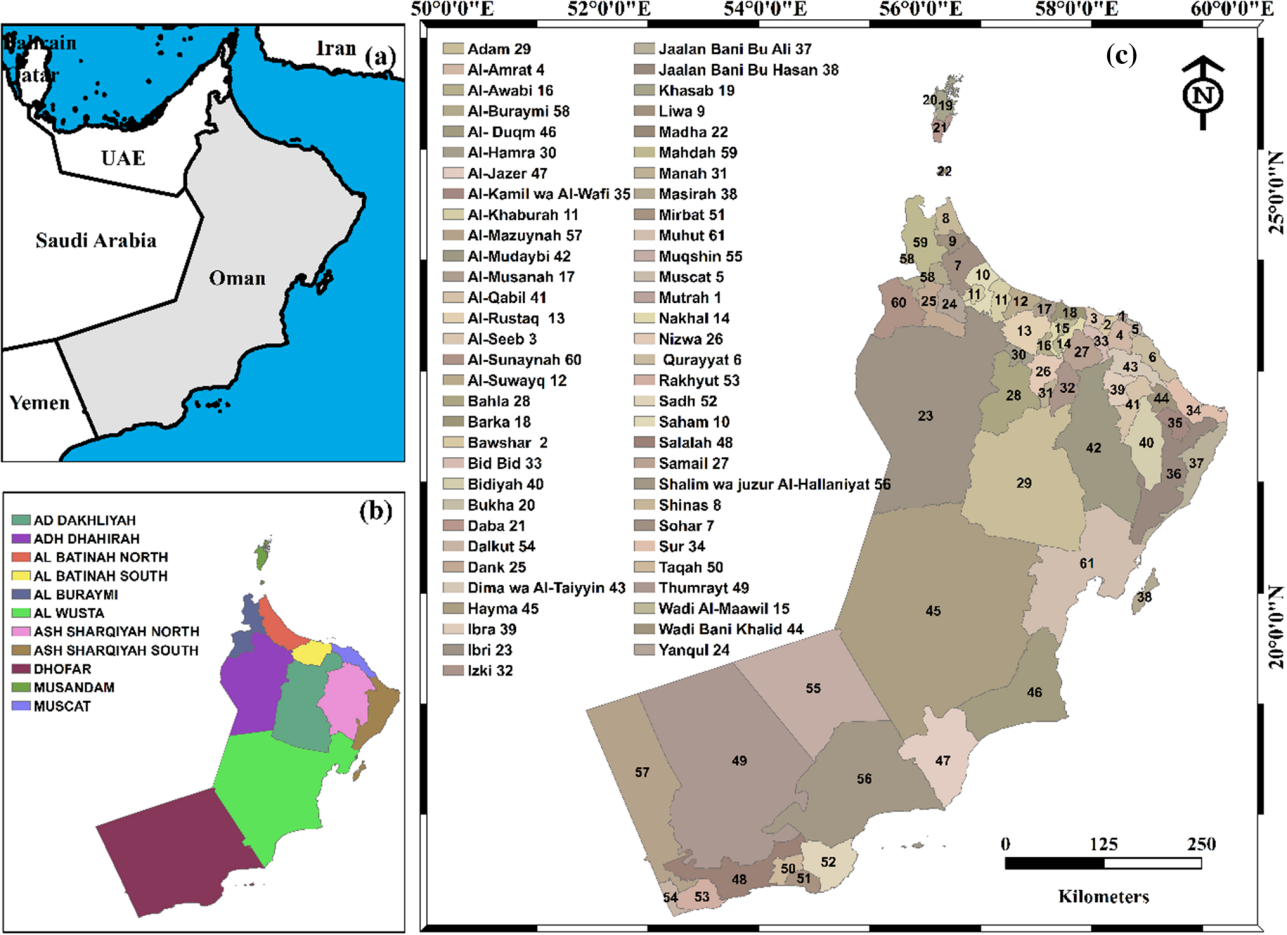
Study area, including: **a** location of Oman; **b** distribution of the 11 governorates in the study area; and **c** wilayats in the study area

**Fig. 2:**
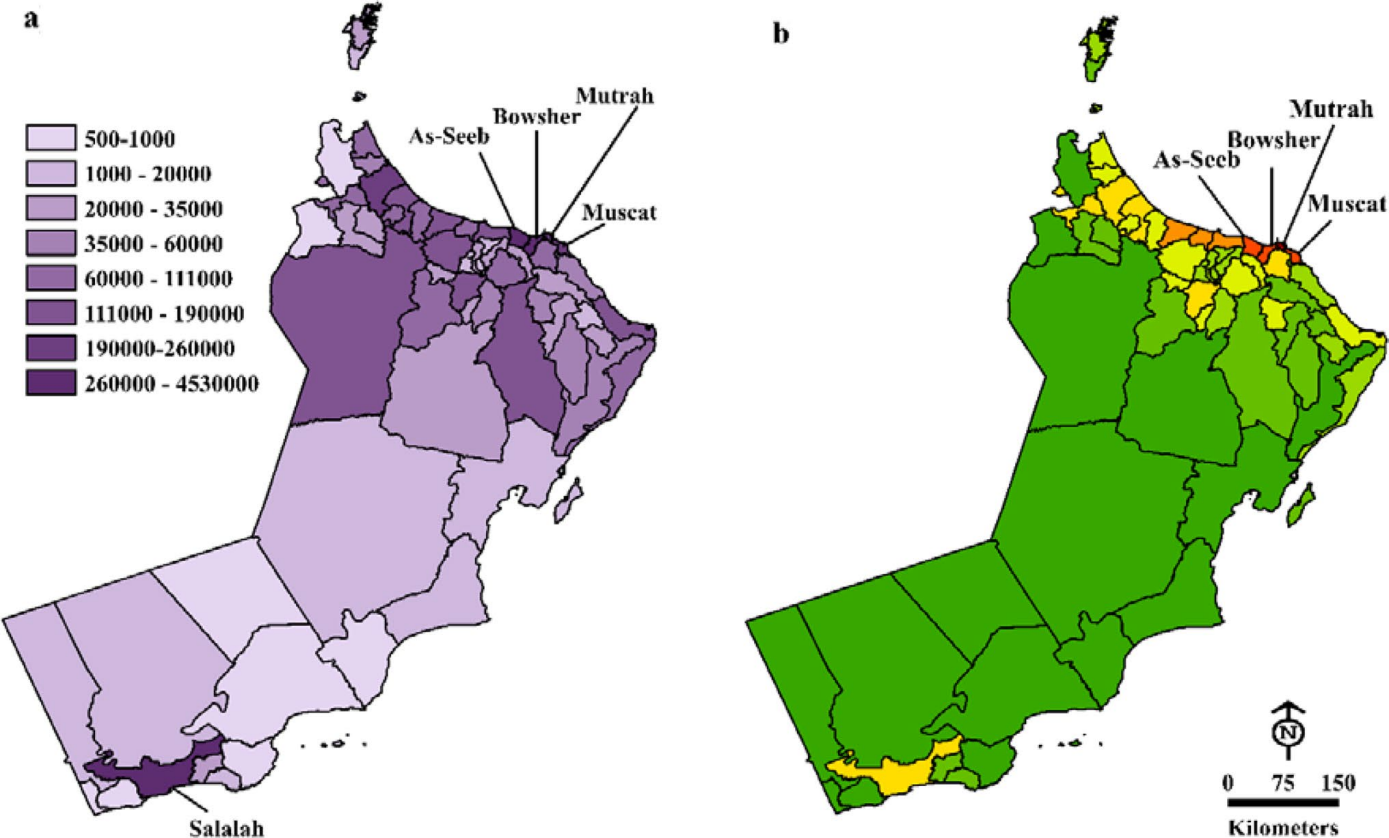
Schematic map showing the distribution of population in Oman in 2019: **a** population distribution in 61 wilayats in 2019; **b** population density (population/km^2^) for each wilayat in 2019. The same scale is used for all maps

Oman occupies the southeast corner of the Arabian Peninsula and is located between latitudes of 16° 40´ and 20° 20´ N and longitudes of 51° 50´ and 59° 40 E. The total area of Oman is 309,500 km^2^ and it has a coastline extending almost 165 km from the Strait of Hormuz in the north to the Republic of Yemen in the south. their are 11 governorates (Fig. 1b) and 61 wilayats in Oman (Fig. 1c). The total population of Oman is 4,617,927 based on the Oman’s National Centre for Statistics and Information data (NCSI 2019). Figure 2 shows the population and population density (population/km^2^) for each province in 2019. Based on Oman’s National Centre for Statistics and Information (NCSI 2019), Bowsher, As-Seeb and Salalah had the highest populations; Mutrah ranked fourth in population but first in population density (NCSI 2019).

### Data Set Description

Real-time data on COVID-19 were obtained using the Tarassud + App (TA +): a mobile application developed by the Oman’s Ministry of Health (OMH). The TA + displays the country status of COVID-19, guidelines, self-reported data, statements and other COVID-19 metadata, including an interactive world map for coronavirus statistics (Ming et al. 2020; Sohrabi et al. 2020). The TA + is updated regularly—on a daily basis—with data on new, existing confirmed cases, recoveries, and deaths through the Omani governorate and local authorities. Although TA + has an application programming interface (API) to extract updated information, official statements are delivered publicly only by the Ministry of Health (MOH). The virus was confirmed to has reached Oman on 24th February following a confirmed positive test for two citizens arrived from Iran.

**Fig. 3:**
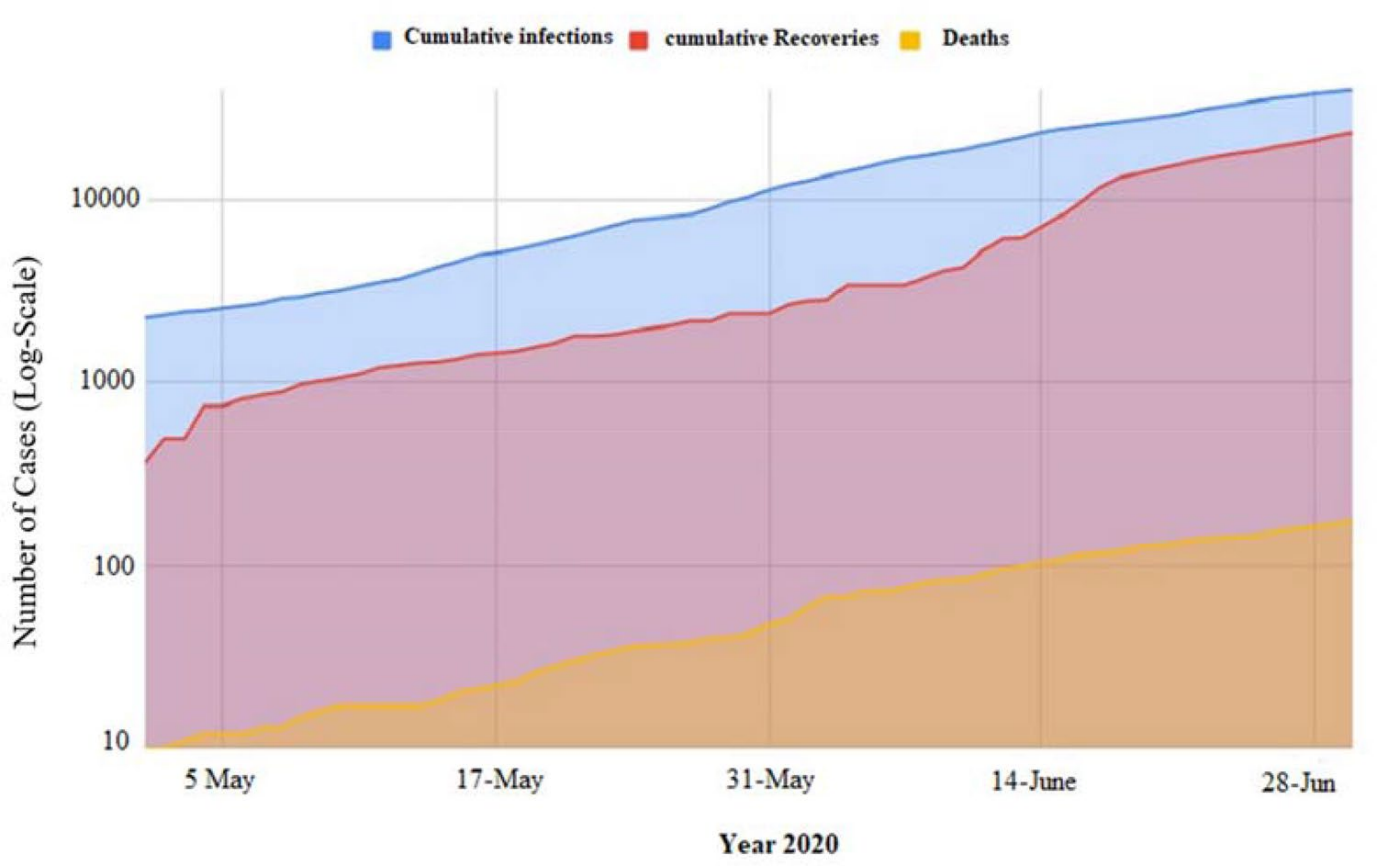
A log–log plot showing the growth in the accumulative, recovered, and deaths cases between 29th April and 30th June 2020 in Oman

**Fig. 4:**
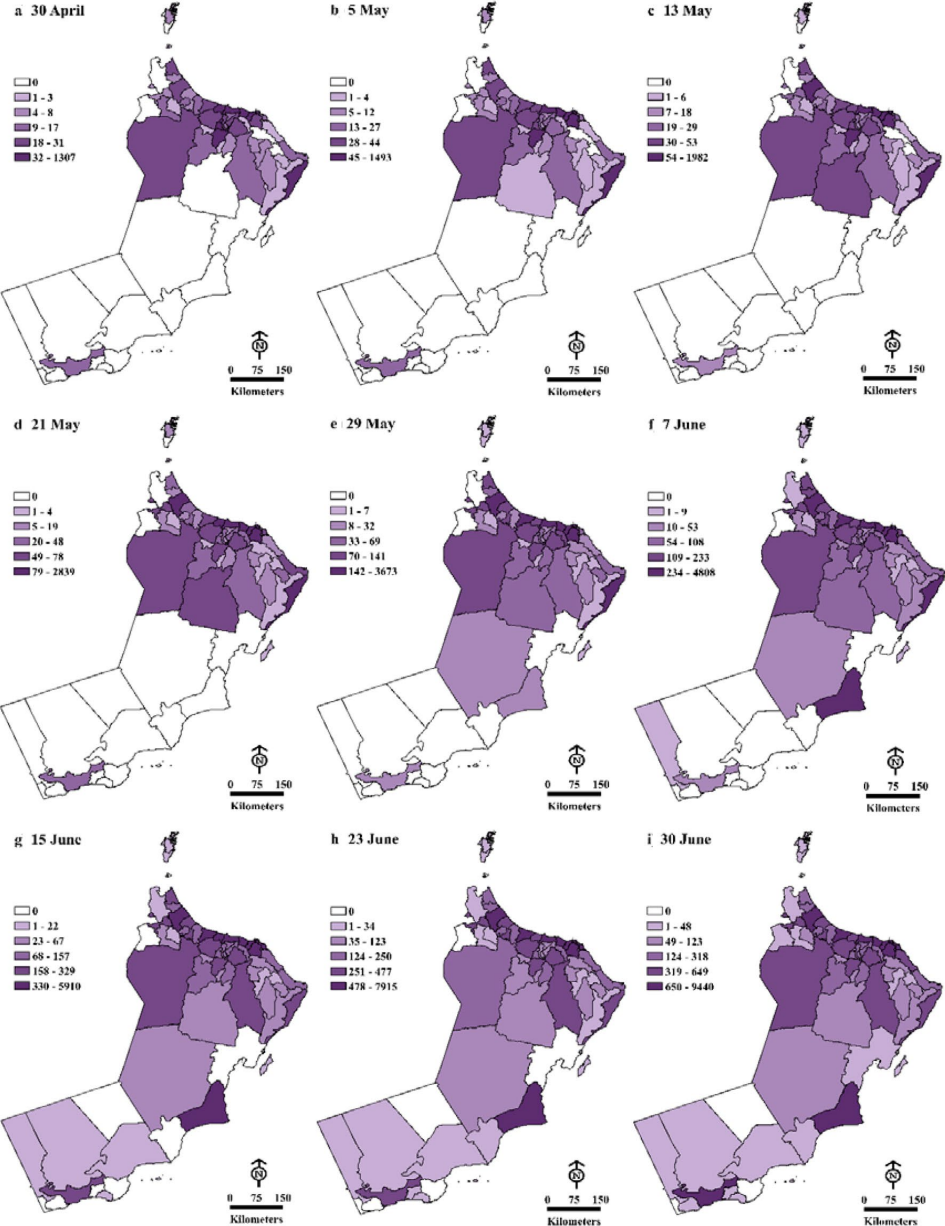
Maps showing the number of cases per week for each wilayat between 29th April and 30th June in Oman

This study deployed daily COVID-19 data for 8 weeks spanning the period between 29th April and 30th June 2020. The early weeks of the spread were not included in this study given the low number of confirmed cases. Even these few cases distributed over a small number of wilayats. Figure 3 illustrates the growth in the accumulative, recovered and death cases between 29th April and 30th June in Oman. However, although the selected study period can be seen as an early stage in the proposed timeline of COVID-19 in Oman, this period corresponded to a sharp increase in the total infected cases across the country. Accordingly, the selected study period gives an opportunity to explore the spatial distribution of COVID-19 in Omani wilayats in a more robust way. The daily data of COVID, combined with relevant spatial data (i.e., coordinates of locations of the cases), were employed in this study. Table 1 lists the different spatial data used in this study and their attributes. Importantly, the data were collected at varying spatial scales (i.e., governorates, wilayats, districts, and even street level). All these spatial data were integrated in a geodatabase in a GIS environment. Figure 4 depicts the spatial distribution of the confirmed cases per each week over the period from 29th April to 30th June 2020. Also, we showed the spatial distribution of COVID-19 cases, but per 100,000 of population in each wilayat. This gives indications on the different hotspots of this pandemic, as a function of the total number of populations in each wilayat.

**Table 1:** Datasets obtained from Oman’s Ministries and Departments

	Variables	Format	Source
1	Population size	GIS Shapefile (polygon)	NCSI
2	Population density	GIS Shapefile (polygon)	NCSI
3	Districts map	GIS Shapefile (polygon)	NCSI
4	Governorates and Wilayats boundaries	GIS Shapefile (polygon)	NCSI
5	Daily COVID-19 data	TA + (MOH)	MOH
6	COVID-19 (validation data)	Excel-Sheets	MOH

The geodatabase and an excel-sheet data coverage such as COVID-19 confirmed cases, validation data of confirmed cases were obtained from the Ministry of Health (MOH), while data on the population and population density were obtained from National Centre for Statistics and Information (NCSI 2019)

### Spatial Analytic Methods

GIS provides a wide variety of tools that allow for determining different spatial statistics of any epidemic risk (e.g. distribution, hotspots, orientation, trajectories of spread, etc.). In this context, we employed GIS techniques to investigate spatial variations of disease incidence, visualize the epidemic information, and spatial tracking of pandemic hotspots over the study period. This is a preliminary, but necessary, step to understand spatial variability of incidence in relation to different environmental, socioeconomic, topographic, and demographic variables and also for a spatiotemporal prediction of regional transmission speed and magnitude in the near future.

#### Calculating Geographic Distribution

The calculating geographic distribution (CGD) is a commonly used approach by epidemiologists to compare disease distributions over days or weeks (Dong et al. 2017). In this study, we employed CGD, using ArcPro 2.5 software, to analyze spatial distribution of COVID-19 in Oman, mainly its centres and tracing. Simply, this method was used to identify the spread of COVID-19 by calculating and mapping hotspots of spread on a weekly basis from 29th April to 30th June. First, for each week, the mean centre of the outbreak was identified for the whole country. This was made based on daily aggregated data for each week. Second, we computed the weighted variation in the distance between each location with confirmed cases and this mean centres. This procedure was implemented using the standard distance tool within ArcPro 2.5 software. To account for changes in the mean centre over the whole study period, we employed the Standard Deviational Ellipse (SDE) method in ArcPro. This tool gives quantitative assessment of changes in the trajectories of COVID-19 hotspots over the study period (Samphutthanon el al. 2014). Statistically, for each week, SDE calculates the standard deviation of any location, represented in x- and y-coordinates, from the mean centre of the pandemic, illustrating these deviations in an ellipse with a diameter up to one standard deviation (Carnes & Ogneva-Himmelberger 2012). This ellipse has a directional axis, expressed in degrees (0–360), which defines the spatial orientation of COVID-19 spread for this specific week (e.g. 90°: east expansion, 180°: south expansion, 270°: west expansion, etc.). This method allows to quantitatively define changes in the trajectory of COVID-19 main centre between the different weeks (Scott and Janikas 2010). These ellipses also give a visual inspection of changes in the trajectories of COVID-19 over time.

#### Spatially Integrated Statistics

To define the spatial patterns of the spread of COVID-19, we employed two well-established geospatial statistics: global Moran’s *I* and *G* test. These statistics are well-non in the GIS literature as powerful tools to understand spatial patterns of any phenomenon, including epidemic risks (Bailey 2001; Getis 1991; Cromley 2003; Bailey et al. 2011; Adegboye et al. 2020). Moran’s *I* and *G* test are measures of spatial autocorrelation of data, allowing to define spatial clustering of COVID-19 incidence and its varying spatial densities. Spatial data are simply described as highly correlated if likely values are spatially close to each other, and conversely defined as independent or random data if no pattern that explains the arrangement of these data can be identified (Naish et al. 2011; Huang et al. 2020; Kang et al. 2020). In GIS, their are different tools that provide a value of Moran’s *I* magnitude, with positive values indicating a tendency toward clustering, while negative values suggest a random pattern of distribution. Other relevant statistics to Moran’s *I* statistic is *Z* score, which quantifies the degree of deviation (i.e., dispersion or clustering around Moran’s *I* value) and *p* value, which gives indications on the statistical significance of clustering outputs. In this context, significant autocorrelation reveals that the value of the variable at a given location depends on the values at neighbouring locations and vice versa. Typically, the global Moran’s *I* value lies within a range of − 1.0 to + 1.0, with values close to -1 suggesting a typically random pattern of COVID-19 spread and values approaching 1 indicating more clustering (Adegboye et al. 2020; Ceylan 2020). According to Prasannakumar et al. (2011), Moran’s *I* is computed, as:

1 $$I = \frac{{N\mathop \sum \nolimits_{i} \mathop \sum \nolimits_{j} W_{i,j} \left( {X_{i} - \overline{X}} \right)\left( {X_{j} - \overline{X}} \right)}}{{\left( {\mathop \sum \nolimits_{i} \mathop \sum \nolimits_{j} W_{i,j} } \right) \mathop \sum \nolimits_{j} \left( {X_{i} - \overline{X}} \right)\left( {X_{j} - \overline{X}} \right)^{2} }},$$

where *N* is the number of COVID-19 cases, *X*_*i*_ is the variable value at a particular location, *X*_*j*_ is the variable value at another location, *X* is the mean of the variable, and *Wij* is a weight applied to the comparison between location *i* and location *j*. This distance-based weight matrix is based on the inverse distance between locations *I* and * j* (i.e.,, 1/*dij*).

Similar to Moran’s *I* test, *G* test is another indicator of spatial autocorrelation, identifying hotspots and local spatial clustering of COVID-19 (Getis & Aldstadt 2004). However, it is inversely related to Moran’s *I* test, as values close to − 1 indicate aggregation of similar values (i.e., clustering), while values close to 1 suggest segregation (i.e,. random patterns). According to Getis and Aldstadt (2004), *G* is computed, as:

2 $$G = \frac{{\sum\nolimits_{i = 1}^{n} {\sum\limits_{j = 1}^{n} {w_{i,j} x_{i} x_{j} } } }}{{\sum\nolimits_{i = 1}^{n} {\sum\nolimits_{j = 1}^{n} {x_{i} x_{j} } } }},\forall j \ne i,$$

where *x*_*i*_ and *x*_*j*_ are attribute values for locations *i* and *j*, *w*_*i*_*,*_*j*_ is the spatial weighted distance between locations *i* and *j*. *N* is the number of locations, $\forall$ *j* ≠ *i* indicates that locations *i* and *j* cannot reflect the same feature.

*G* test commonly returns four values: observed general *G*, expected general *G*, *Z* score, and *p* value (Getis and Aldstadt 2004). Further details about the computation of these statistics are outlined by Getis and Ord (2010). First, we looked at the *p* value of this statistic. If it is small and statistically significant, this suggests that their is a spatial clustering of the cases. Otherwise, their is a random distribution of the cases. If *p* value suggested a clustering, we look at the sign of *Z* score. A positive sign (i.e., observed General *G* index is larger than the expected General *G* index) indicates that higher values of COVID-19 cases tend to be clustered over the study domain. Rather, a negative sign of *Z* score (i.e., the observed General G index is smaller than the expected index) suggests that lower cases of COVID-19 tend to be grouped (Getis and Ord 2010).

Getis-Ord $G_{i}^{*}$ is another test commonly used in hotspot analysis in GIS. Similar to *G* test, it provides two measures: *Z* score and p value. Both statistics indicate whether the highest/lowest numbers of COVID-19 cases tend to be spatially dependent (i.e., clustering) (Huang et al. 2020; Huling et al. 2020). Specifically, resultant *Z* score informs where locations with either high or low incidence tend to be clustered over space. Importantly, according to Getis-Ord $G_{i}^{*}$, for any location to be considered as a significant hotspot of COVID-19, other locations in the neighbourhood should exhibit high incidence of COVID-19 as well. Accordingly, the local sum of cases for any specific point and its neighbours is compared proportionally to the sum of cases for all points over space. For statistically significant positive *Z* scores, the larger the *Z* score is, the more intense the clustering of high values (hot spot). A statistically significant *Z* score is determined when the local sum is much different than the expected local sum. Following this approach, a *Z* score is assigned to each location over space. For statistically significant positive *Z* scores, higher *Z* score suggests more clustering of higher number of cases (i.e., hot spot). In contrast, statistically significant negative and smaller *Z* scores indicate more clustering of lower incidence of COVID-19 (i.e., cold spot) (Huling et al. 2020). Herein, we calculated $G_{i}^{*}$ statistic to analyze spatial clustering of COVID-19 cases for each week independently for the period from April 29 to June 30 and define the corresponding hotspots and cold spots sites. Herein, $G_{i}^{*}$ is computed, as:

3 $$G_{i}^{*} = \frac{{\sum\nolimits_{j = 1}^{n} {w_{i,j} x_{j} } - \overline{X}\sum\limits_{j = 1}^{n} {w_{i,j} } }}{{s\sqrt {\frac{{\left[ {n\sum\nolimits_{j = 1}^{n} {w_{i,j}^{2} } \left( {\sum\nolimits_{j = 1}^{n} {w_{i,j} } } \right)^{2} } \right]}}{n - 1}} }},$$

where *N* is the number of COVID-19 cases, *X*_*i*_ is the variable value at a particular location, *X*_*j*_ is the variable value at another location, *X* is the mean of the variable, and *Wij* is a weight applied to the comparison between location *i* and location *j*. This distance-based weight matrix is based on the inverse distance between locations *i* and *j* (i.e., 1/*dij*).

## Results

### Spatiotemporal Orientation and Shifting

#### Weighted Mean Center (WMC)

Figures 6 (a small circle highlighted in green) and 7 illustrate the weekly change in the WMC of COVID-19 infections from 29th April to 30th June 2020. During this 9-week phase, the *x*-coordinate of the mean centre of COVID-19 in Oman moved many times. Overall, the tracking change results revealed that the centers of COVID-19 outbreaks moved or spread to the northwest and southwest of Oman. Specifically, the mean center was initially located at 58.22° E, 23.41  N, but an animation of the vicissitudes described a transfer in the WMC over time. For instance, on 5 May 2020 the WMC was placed at 58.23° E, 23.43° N, but had shifted to 58.23° E, 23.46° N, about 3 km northwest, by 13 May 2020 (Table 2).

**Table 2:** The centre of COVID-19 weighted by the number of cases for each wilayat over the 9-weeks period (highlighted in green in Fig. 5)

Date	X-Coord	Y-Coord	km^2^
29-Apr-20	58.255212	23.419215	0
5-May-20	58.239013	23.431584	2.29
13-May-20	58.240718	23.433133	0.490
21-May-20	58.234877	23.459678	3
29-May-20	58.209793	23.459678	3.32
7-June-20	58.234877	23.459678	4.11
15-June-20	58.106908	23.312946	13.44
23-June-20	58.047374	23.297791	6.4
30-June-20	57.97499	23.266568	8.14

**Fig. 5:**
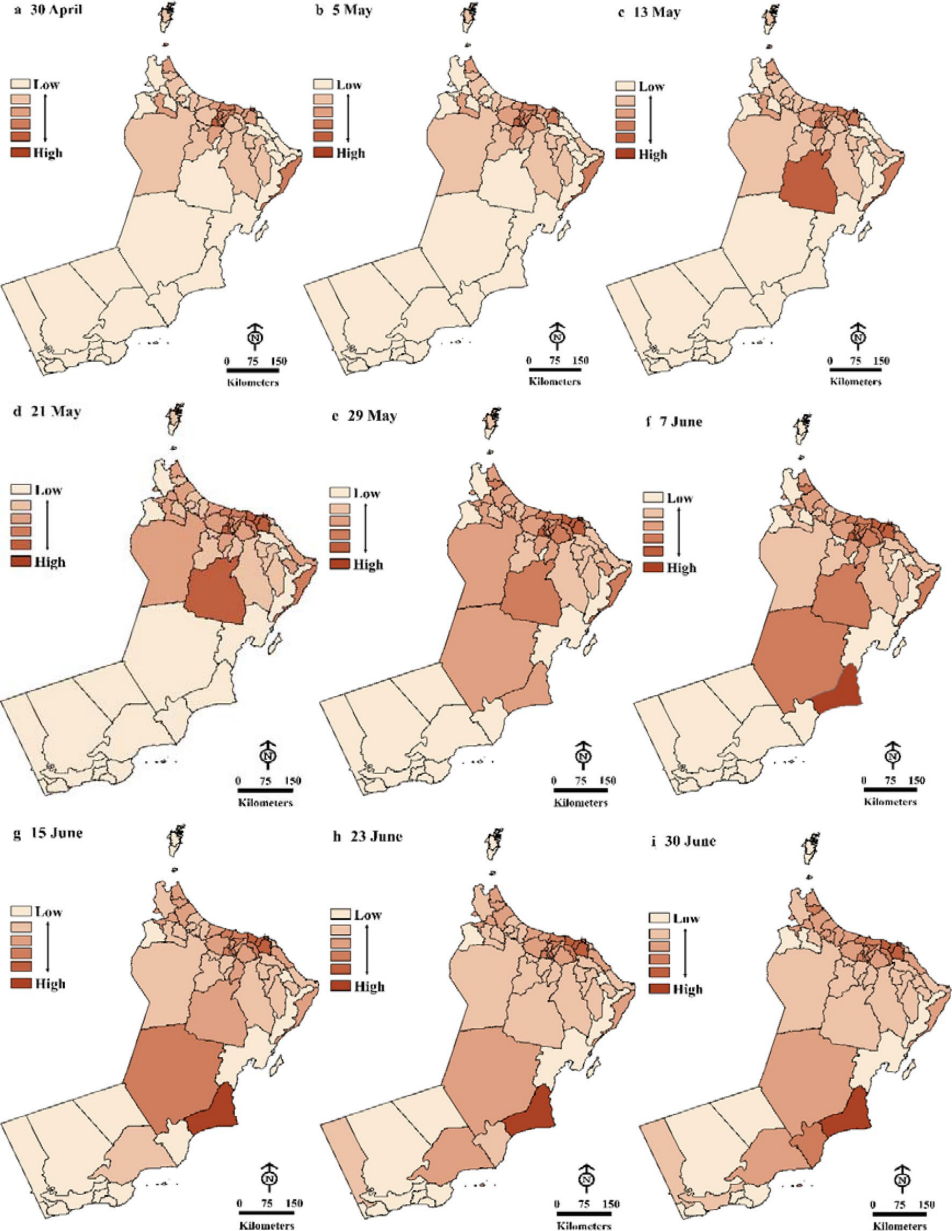
Maps showing COVID-19 count per 100,000 and week for each wilayat between 29th April and 30th June in Oman

#### Directional Distribution

**Fig. 6:**
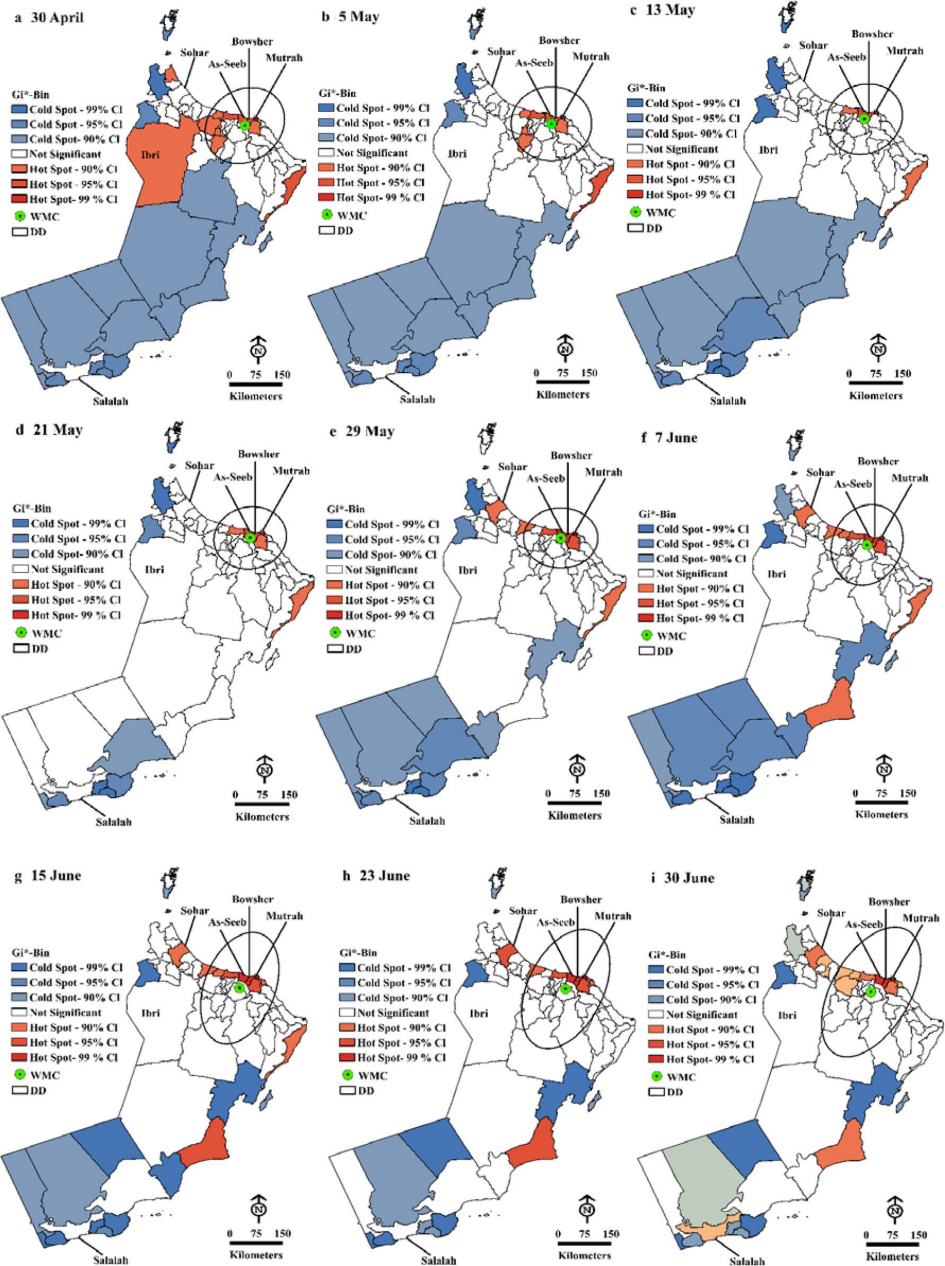
Clustering of COVID-19 (using the infection rates for each wilayat as the attribute value). Locations with similarly high numbers of COVID-19 (hotspots) are shown in dark green. COVID-19 rates coded by Gi* statistics display the prevalence of COVID-19 based on weekly data from 29th April to 30th June 2020. The centre of COVID-19 is weighted by the number of cases and over each wilayat over the 9-weeks period (highlighted with green). Standard deviational ellipses of COVID-19 infections distribution in a study area over the 9-week period from 29th April to 30th June 2020 (highlighted in black)

**Fig. 7:**
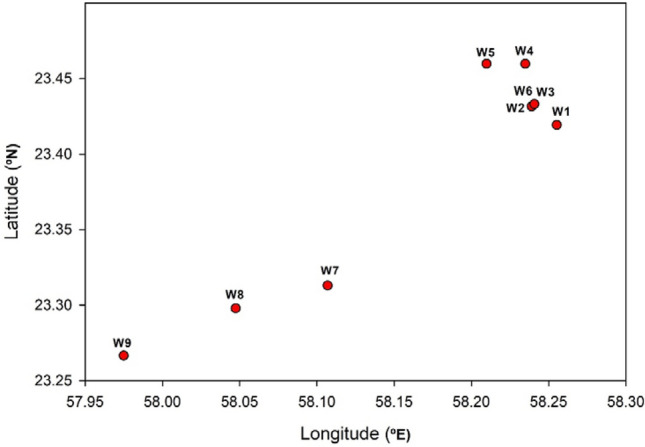
Shift in the weighted mean center over the study period. The centre of COVID-19 is weighted by the number of cases for each wilayat over the 9-weeks period (highlighted in green in Fig. 6)

Directional distribution (DD) analysis indicated that the trend of the COVID-19 cases shifted from northeast to northwest (Fig. 6). As noted, during the 9-week period, the main hotspots of COVID-19 were placed primarily in northern Oman (wilayats of Mutrah, Bowsher, and As-Seeb). Getis-Ord $G_{i}^{*}$ coefficient suggests that these three wilayats were defined as hotspots at a significance level of 99% (*p* < 0.02). While wilayat of Ibri was defined as a hotspot (*p* < 0.05) in the first week of the study area (30th April–4th May), it rapidly converted to a wilayat with non-significant clustering of COVID-19 cases in Oman (*p* > 0.05). Rather, few wilayats on the eastern coast were defined as significant hotspots in Oman. Notably, southern and most of inner wilayats were classified wither as cold spot regions or regions with non-significant clustering of COVID-19 incidence over the study period. It should be indicated that the virus expanded over the country, but the rate of westbound spread is noteworthy. Ellipses changed in size and shifted from the northeast to the northwest and southwest during the study period. Table 3 displays the axis lengths, rotation, and area of each ellipse. The size of the ellipses increased and decreased over time. The orientation coincided with the spatiotemporal agglomerate characteristics such as population and population density, indicating that the spread of COVID-19 infections exhibited both orientation and direction and showed a spatiotemporal trend in the 9 weeks from 29 April to 30 June 2020. Thus, the extent of the SD varied from week to week. For example, the width area of the ellipse was 194 km and its length was 227 km on 29 April 2020, while on 29 May 2020, it was 176 km in width area and 184 km in length (Fig. 6 and Table 3). Although Table 3 shows that the east axis moved 40 km west by the end of the study phase, regardless of increase or decrease over time, the distribution relative to the mean centre was more concentrated between 57.97° and 58.25° E (see Fig. 7).

**Table 3:** Changes in the DD (1 SD) from the weighted mean centre of COVID-19 infections over the 9-week period

Date	Length	Weight	Area (km^2^)	Rotation
29-Apr	227	195	34,862	50
5-May	225	194	34,487	55
13-May	211	192	31,921	53
21-May	196	181	24,253	74
29-May	175	188	22,384	88
7-June	176	184	25,391	90
15-June	326	194	47,966	18
23-June	360	211	55,889	23
30-June	399	213	65,624	27

### Spatiotemporal Spread

**Fig. 8:**
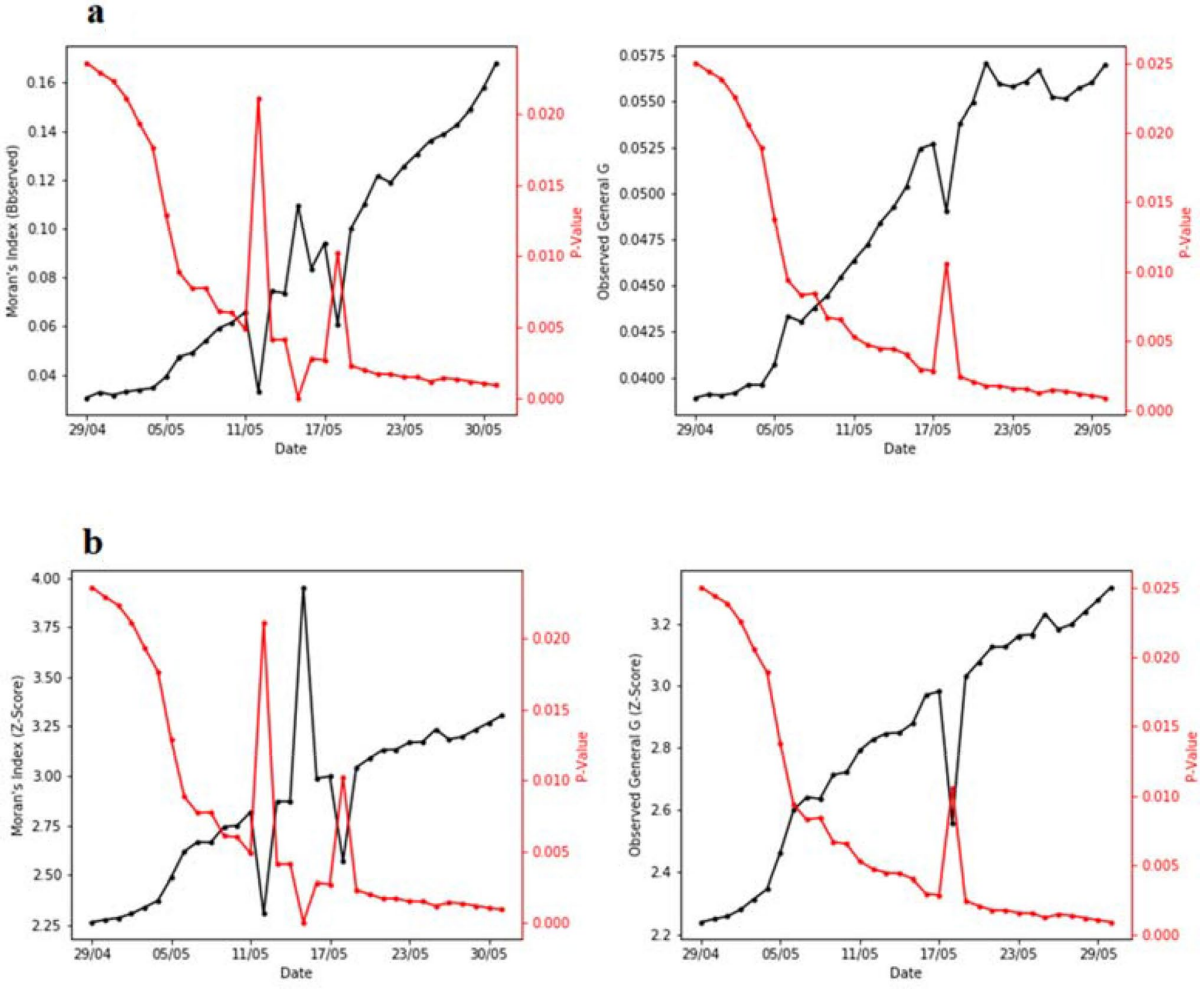
**a** Autocorrelation (Moran’s *I*) and observed General G statistic marked with black, while (Moran’s *I*, and G test *p* values) is marked with red; Fig. 4**b**. Autocorrelation (Moran’s *I*) and observed General *G* statistic marked with black, while (Moran’s *I*, and *G* test *Z* score) is marked with red

The global Moran’s *I* statistic showed that COVID-19 cases in datasets (numbers of confirmed cases) were clustering throughout the study. All of the Moran’s *I* and *Z* scores were well above the 2.25 threshold (a confidence level above 95%), ranging from 2274 cases on 29 April to 40,070 cases on 30 June 2020. In regions wherever their existed higher numbers of cases, neighboring wilayats inclined to have analogous number of cases. Our results showed a significant spatial autocorrelation, indicating that COVID-19 rates between wilayats were positively and significantly spatially related (clustering with distances) from 29 April to 30 June 2020 (see Fig. 8). It appears that the pattern of COVID-19 becomes more clustered over time in the study area; it could indicate that the disease is spreading less rapidly. Similarly, the Moran’s *I* and G test statistics indicated positive relationships between COVID-19 rates and population density (Moran’s *I* = 0.276, *Z* score = 7.274, *p *value = 0.0001 and *G* = 0.0002, *Z* score = 7.506, *p *value = 0.0001, respectively). Likewise, positive relationships were found between COVID-19 rates and total population (Moran’s *I* = 0.204, *Z* score = 4.367, *p *value = 0.0001 and *G* = 0.00007, *Z* score = 3.946, *p *value = 0.00007, respectively).

### Spatial Clustering

The results are presented every week from 29 April to 30 June 2020 in Fig. 6. They indicate hotspot areas with significantly high infection rates findings. However, our results showed varying rates of infections, and the pattern of risk changed with time (see Fig. 6): some wilayats had more or fewer infections than others. For example, wilayats such as As-Seeb and Bowsher in the Muscat Governorate, in particular, were considered medium-risk areas from 5 to 21 May 2020 but were identified as high-risk areas (hotspot-95% confidence) from 29 May to 30 June 2020 the conditions that Z score higher than 3.50 (hotspot-95% confidence). Another example, wilayat Sohar in Al-Batinah North Governorate, was identified as a non-significant area from 5th May to 21st June 2020, but a low-risk area (hotspot- 90% confidence) from 29th June to 15th June and a medium-risk area on 23rd June 2020 (see Fig. 6). Based on the COVID-19 level for the 9 weeks, we were able to identify willayat Mutrah (Muscat Governorate), as the one with the highest rates of infection in the whole country (*Z* scores ranging between 7.5 and 5.2 from 30 April to 7 June 2020), whereas it classified as a second highest rates from 15 to 30 June 2020. This approach intimates that epidemiologists can understand illness case clusters when they factor in spatiotemporal characteristics.

## Discussion

This study applied a different of spatiotemporal and statistical methods including as a CGD, pattern, and clustering analysing, all of which are important to understand the spread of COVID-19 in Oman from 29 April to 30 June. The first approach applied in this study was to analyze the geographic distribution of COVID-19. The weighted mean centre changed throughout the study phase (see Fig. 6, a small circle highlighted in green). The capability to ascertain a weighted COVID-19 centre is valuable for tracing variations in the distribution coz it acts well when investigating the distribution of values related to an area. These results revealed dynamic areas of infections. One probable reason for the continued increase in COVID-19 infections and the vicissitudes and shifts between wilayats stated in this research is the imperfection of the control strategies currently being practiced restricting the spread of the virus since the first case was identified on 24 February 2020. Another potential reason could be that COVID-19 community transmission in certain regions may have been overlooked or certain regions were not classified as risk areas. Furthermore, the current data and limited study period may not be enough to discover the valid reasons for the high concentrations of COVID-19 infections.

Although control measures such as home quarantine, social distancing and wearing masks have been implemented across Oman, the number of confirmed cases continues to increase steadily. Tracking the changes in the distribution of infections will halp epidemiologists and authorities in Oman to predict where the next hotspot will appear, and thus attempt to prevent it by ordering lockdowns before the rate of infection increases. DD indicates this trend, coz it is a confidential statement and renders results based on an analytical method rather than merely a distinct representation of maps (see Fig. 6, ellipses highlighted in black). The ellipses of COVID-19 coincide with the population numbers and densities features of affected areas (see Fig. 6, ellipses highlighted in black). This may be due to the wilayat of Mutrah being a historic and busy trade centre—it also includes Mina Sultan Qaboos, Oman’s main port (Alkamali et al. 2017); Bowsher, a new town which is the location of various government offices and organisations; and As Seeb, an ancient town surrounded by a number of farms, a popular summer resort and industrial zone with high reliance on foreigners. All these circumstances may have facilitated the spread of COVID-19 in the area.

This research also aimed to identify the spatiotemporal patterns of COVID-19 in Oman. Moran’s *I* was applied to identify clusters using attribute values and locations of COVID-19. This is typically done with polygons containing a summary statistic, such as COVID-19 case rates, census data or population density data. It is critical to perceive that the autocorrelation cannot be used to identify clusters, as shown in spatially integrated charts such as Fig. 8. It indicates whether the patterns of values over the study area are distributed in an assembled, irregular or dispersed way (Zhang and Zhang 2007).

Our results showed that the average difference between neighbouring features is less than those between all the features; this remained true for the values which appeared to cluster throughout the study period, from 29 April to 30 June 2020. However, the Moran’s *I* autocorrelations did not identify features of the variables (e.g. the distance between values, population or population density) as high and low cluster values, as we applied the G test for this purpose. theirfore, the autocorrelation and G test are essential methods to identify robust spatiotemporal patterns in the associations between factors and COVID-19 infections; however, they only considered the distribution of an individual element in a single layer at a time (see Fig. 8). It is hard to conclude whether robust or weak associations are enhancing higher or lower spatially ghettoized. For this, we applied Gi* to map hotspots and cold spots across the country. Mapping the hotspots and cold spots of infection was the third step in understanding how it spread. By applying procedures such as the Gi*, we were capable of recognizing spatial areas showing disease incidences with a higher certainty (Fig. 6). The $G_{i}^{*}$ maps showed potential epidemics as well as explaining the underlying origin of infection (Fig. 6). These maps also allowed us to compare places based on quantities (Baah et al. 2015) and to identify which sites meet our criteria to understand the relationships between locations in the study area.

Mapping the hotspots and cold spots of infection can be also used to map health statistics to compare the quality of health care in Oman (McLafferty 2003). The Ministry of Health in Oman and other public officials can apply classification maps to see how and where health care varies. Our results presented hotspots of the geographical distribution of COVID-19 from 29 April to 30 June 2020 (see Fig. 6). The disease has prominent regional properties in terms of geographical distribution among 61 wilayats, with significant spatiotemporal agglomeration. It is vital to note that the infection was initially concentrated in wilayat Mutrah; it spread first to neighbouring wilayats, particularly As-Seeb and Bowsher in the Muscat Governorate, and then throughout the country.

Analyzing the possible causes such as professions and industries most at risk, the density of living arrangements (not simply population density), of COVID-19 infections is halpful to hazard supervisors in deciding where to concentrate their resources. This research can significantly halp make the country-wise healthcare policies by applying GIS tools to manage temporal assessments of pandemic diseases such as COVID-19. Thus, wise healthcare policies are required to provide developed COVID-19 monitoring and provide more useful intervention and control of the novel coronavirus in Oman.

## Conclusion

Based on the data of COVID-19 in Oman from 29 April to 30 June 2020, in this study, the spatial CGD, Moran’s *I*, General- *G*, and $G_{i}^{*}$ statistics were adopted to deduce that COVID-19 has had a significant spatial correlation and clustering in Oman. Although the global Moran’s *I* and *G*-statistic identified strong spatial patterns of the COVID-19 in the relationships between variables, these approaches only considered the distribution of single layers at a specific time. It was hard to determine whether strong or weak relationships were more and less spatially segregated. Using $G_{i}^{*}$ (hotspots and cold-spots analysis), we were able to identify which spatial districts showed a high likelihood of infection events. Therefore, links between COVID-19 hotspots, cold-spots, density, density, presence, or absence, can be useful in future studies to investigate their correlations, such as ecological, climatological, and socioeconomic variables. The epidemic situation in wilayat, such as Mutrah, As-Seeb, and Bowsher in the Muscat Governorate, is more severe, and the current transmission still presents an increasing trend. Therefore, the transmission capacity of COVID-19 in other wilayats in Oman is strong. The spatiotemporal risk details exhibited in this research indicate that the temporal hazard model − based on weekly infection rates produces a better understanding of changes. Remaining to develop the prevailing COVID-19 monitoring regime's effectiveness is vital to give more precise, comprehensive monitoring data. In turn, it will provide useful strategies for enhancing the transmission disease surveillance system and controlling interventions in any effected region. GIS can be used to map the disease's occurrence against multiple parameters, including demographics, the environment, geography, and past incidents to understand the origin of outbreaks, spread patterns, and intensity, which in turn supports the implementation of control, preventive and surveillance measures.

## Supplementary Information

Below is the link to the electronic supplementary material.

Supplementary file2 (DOCX 815 KB)
