# Supplementary material for: Spatiotemporal Assessment of COVID-19 Spread over Oman Using GIS Techniques
Source: Earth Syst Environ. 2020 Dec 8;4(4):797–811. doi: 10.1007/s41748-020-00194-2 (PMC7721548; doi:10.1007/s41748-020-00194-2)

Supplementary 1

Clustering of COVID-19 (using the infection rates for each wilayat as the attribute value). Locations with similarly high numbers of COVID-19 (hotspots) are shown in dark green. COVID-19 rates coded by Z-score for Gi* statistics display the prevalence of COVID-19 based on weekly data from 29th April to 30th June 2020. The centre of COVID-19 is weighted by the number of cases and over each willayat over the 9-weeks period (highlighted with red). Standard deviational ellipses of COVID-19 infections distribution in a study area (1 SD) over the 9-week period from 29^th^ April to 30th June 2020 (highlighted in blue).


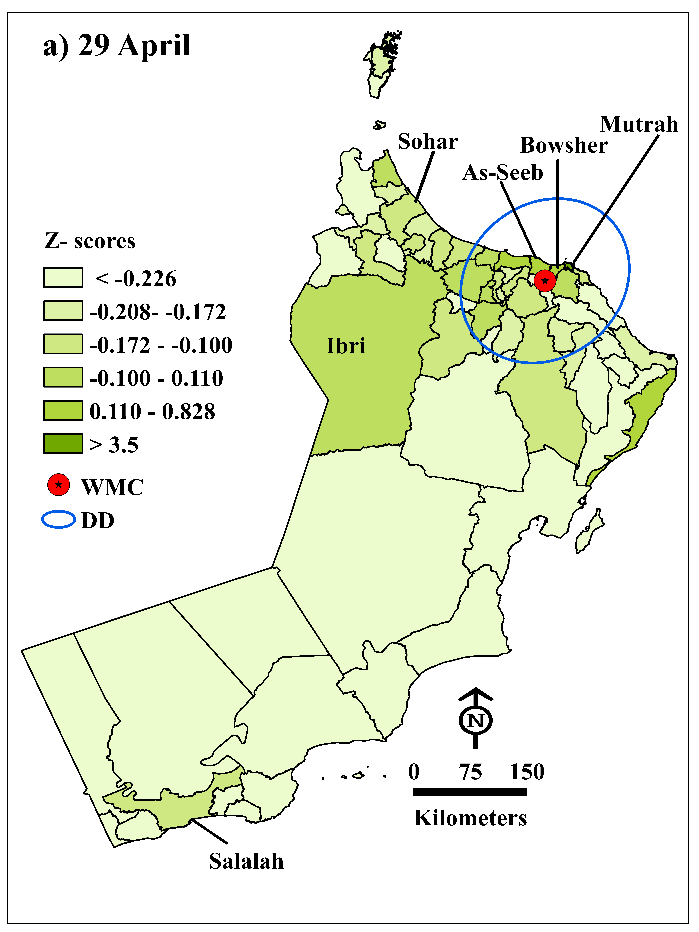

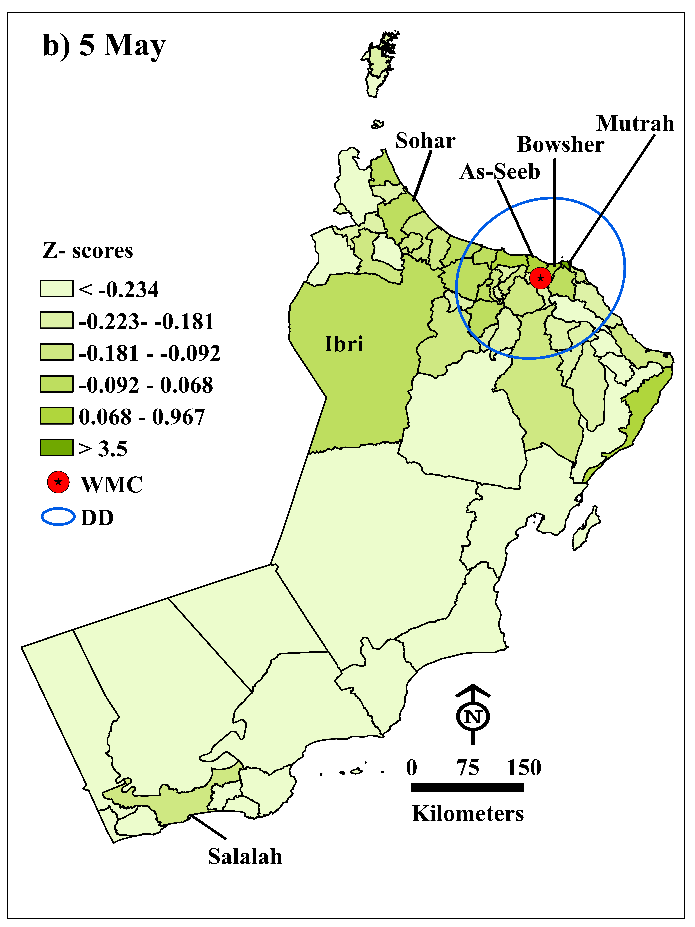


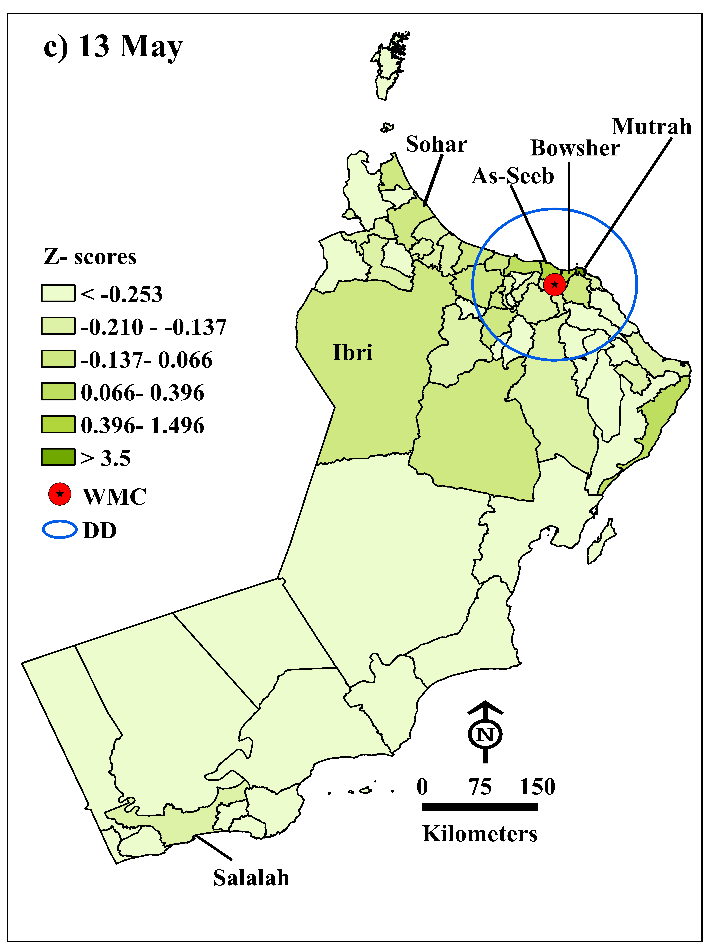

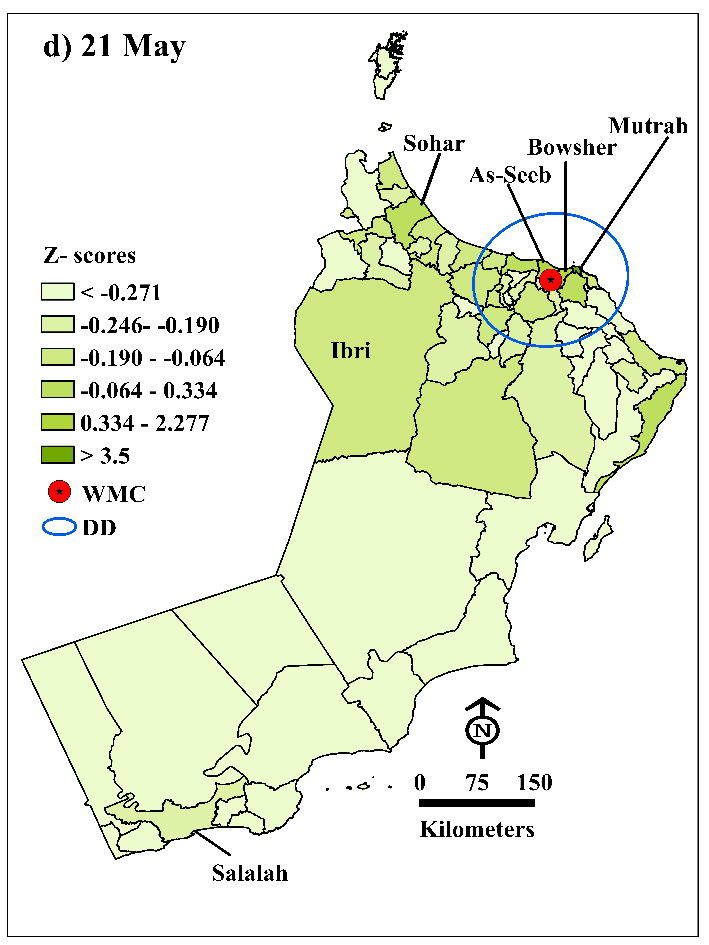


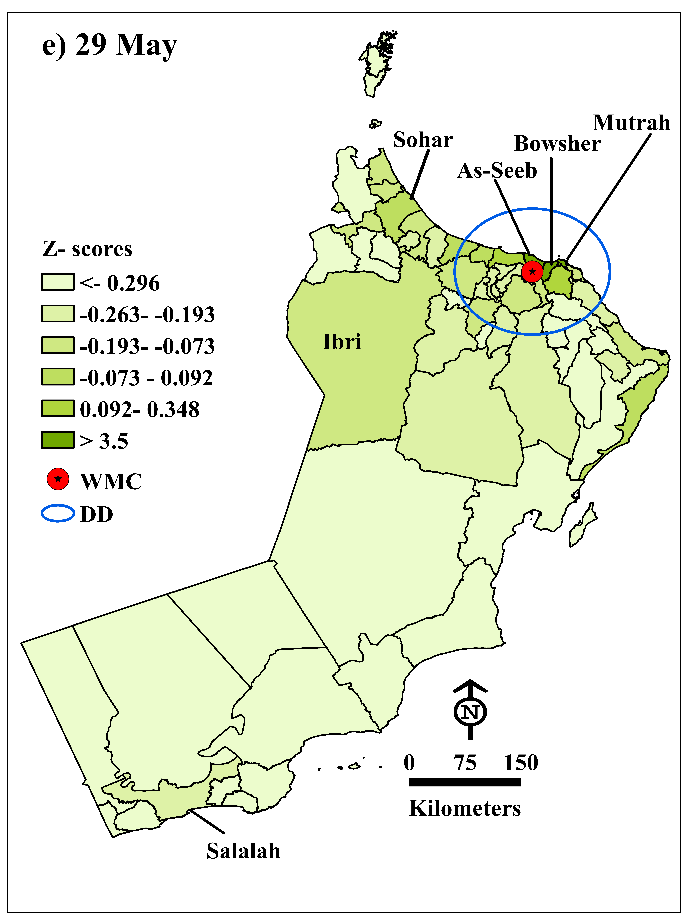

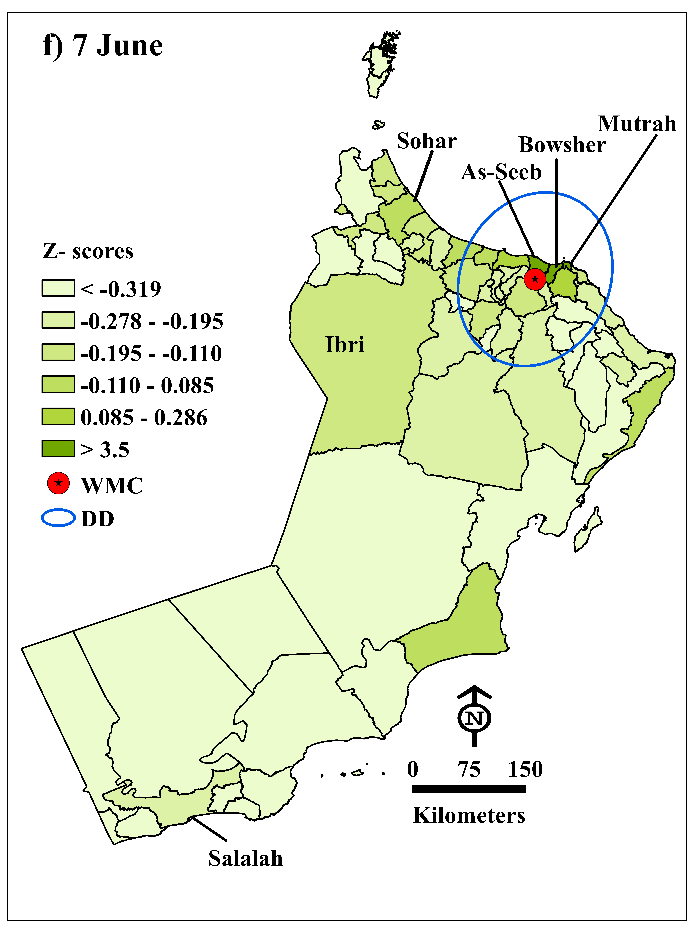


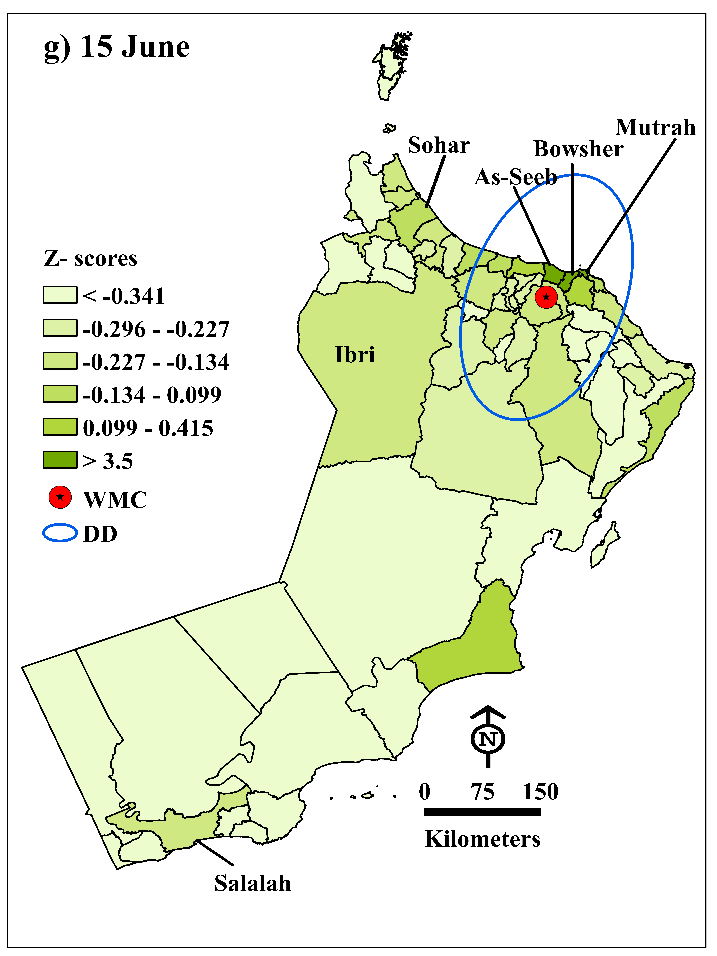

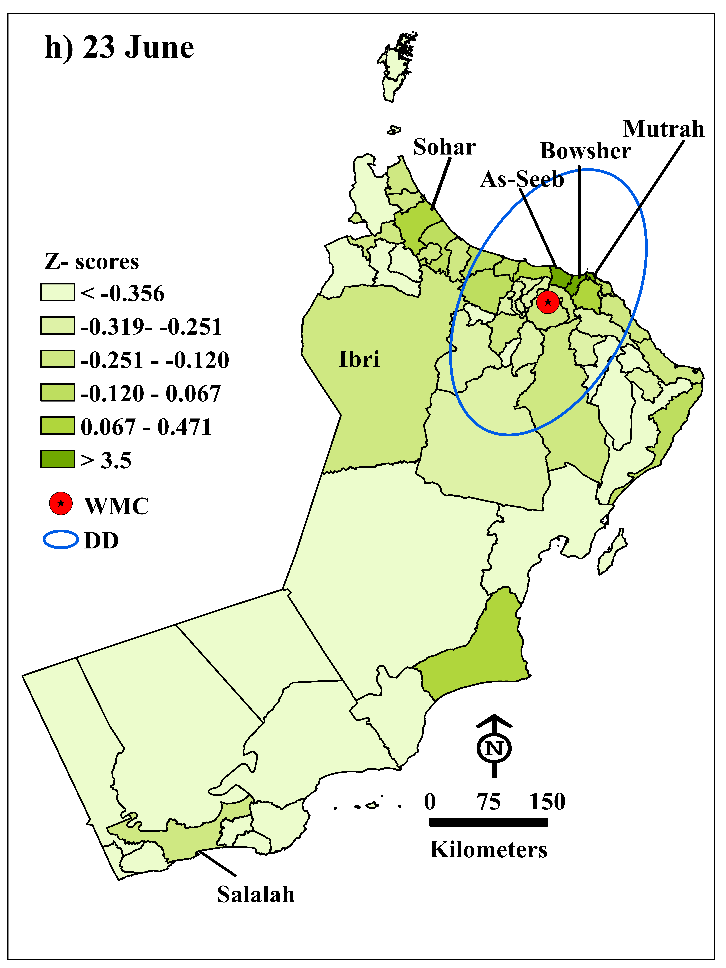


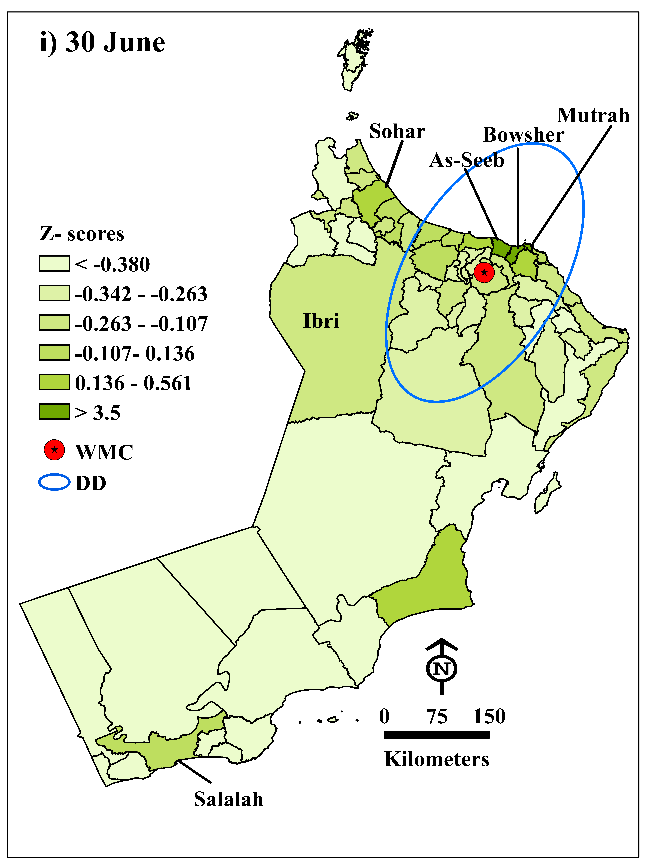


Supplementary 2. The global autocorrelation (Moran’s *I*) and observed General G statistic.


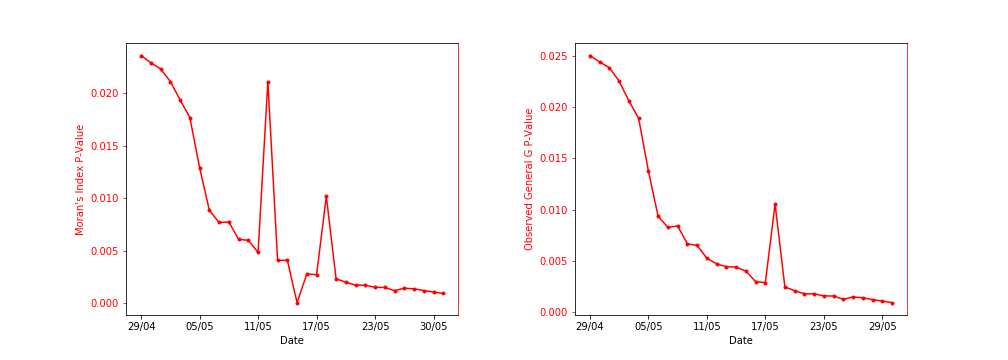


Supplementary 3. Error bars of the Morn’s Index and Observed General G-Test.


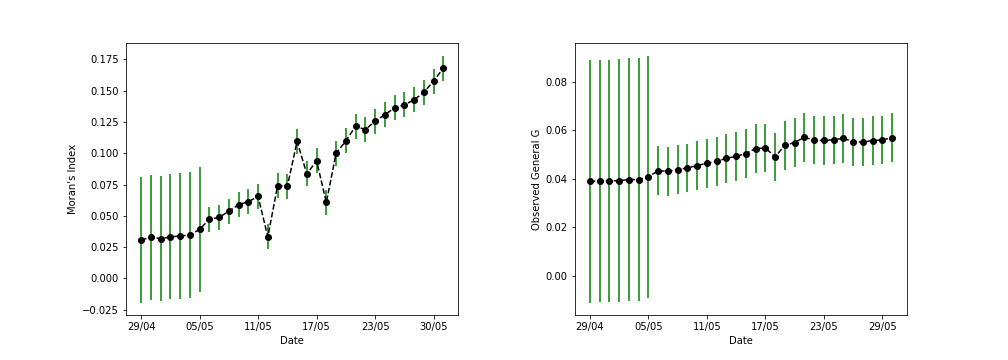

Supplement: Supplementary file 1 — Supplementary file2 (DOCX 815 KB) [file 41748_2020_194_MOESM1_ESM.docx]
